# Book Review: Language Education in Digital Spaces: Perspectives on Autonomy and Interaction

**DOI:** 10.3389/fpsyg.2021.812070

**Published:** 2021-12-07

**Authors:** Kiyana Zhaleh

**Affiliations:** Department of English Language and Literature, Faculty of Persian Literature and Foreign Languages, Allameh Tabataba'i University, Tehran, Iran

**Keywords:** technology-mediated language learning, learner autonomy (LA), learner-directed learning, critical digital literacy, social justice & equity

Carolin Fuchs, Mirjam Hauck, and Melinda Dooly (eds.) (Cham: Springer Nature Switzerland AG) 2021, ix+230 pages, ISBN: 978-3-030-74958-3

The inevitable permeation of technology into all aspects of our life has transformed the way language education is conceived and enacted and put a spotlight on the necessity of promoting *learner autonomy* in digital spaces, especially during this present global pandemic, which has mandated a shift to remote learning and teaching since Spring of 2020. Consequently, Carolin Fuchs, Mirjam Hauck, and Melinda Dooly's edited compendium, entitled *Language Education in Digital Spaces: Perspectives on Autonomy and Interaction*, is a timely contribution that not only underscores technology-enhanced language learner autonomy within/beyond formal instructional contexts but also considers the growing spread of multicultural, multimodal, and intricate online learning environments. This book is indeed enlightening as it delineates how attending to agency, digital literacy, teacher-student interactions, self-regulation, and social justice contributes to creation of autonomous and empowered learners who make informed choices for their learning and flourishment.

The book initiates with a concise introductory chapter written by the editors, depicting its theoretical underpinnings, structure, and main objectives. They nicely foreground the relational and social nature of language use for highlighting the prominence of *autonomy* and *interaction* in remote learning, which are, respectively, lynchpins to Positive Psychology and Instructional Communication theorizing in language education. The remaining chapters form a collection of nine theoretically-rich but practice-oriented empirical studies that, by adopting a wide range of epistemological and analytical frameworks, amalgamate novel findings on how to promote technology-mediated learner autonomy in a spectrum of socio-institutional/geographical instructional contexts (e.g., entirely online interactions, hybrid/blended language learning environments, single-learner multiple app contexts, or multi-player game-based environments) through creative employment of digital affordances (e.g., virtual/telecollaborative exchanges, blogs, apps, self-access technologies, social media, and gaming).

Chapter 2 reported how in- and out-of-class opportunities for using interactional affordances like Facebook resulted in teacher- or student-directed learning among four English university learners in Hong Kong and helped them to become socially responsible when developing identities and communicating in digital spaces. Chapter 3 presented useful findings on how peer comments, teacher scaffolding, use of (a)synchronous digital tools, and social engagement facilitated L2 Spanish students' motivation, self-regulation, and individual/collaborative learning during a flipped-learning course. Chapter 4 underscores the importance of telecollaboration in an exam-based experiential learning context for promoting 55 Chinese university students' self-regulated learning, autonomy, and engagement against the backdrop of appropriately incorporating tasks into assessments and assignments.

Chapter 5 enlightens readers' perspectives about how two groups of Polish and Spanish EFL student teachers' autonomy to engage with semiotic resources can be fostered through a task-based approach to multimodal literacy development in virtual exchange settings. Chapter 6 is similarly insightful for teachers and teacher educators as it presented how a 16-year continuous telecollaboration and involvement in a teacher education program gradually increased former teacher education students' self-direction, self-regulation, and agency in how to create learning environments for their students employing telecollaboration.

Chapter 7 explored reflections of learners and teacher candidates in Colombia regarding their synchronous virtual exchanges in Zoom. Results revealed that through reflection on their interactions with learners, teacher candidates were empowered to engage with the discourse and expressed increase in linguistic/cultural knowledge while learners' reflections revealed their being afforded the opportunity to engage in interactions with teachers to develop confidence, proficiency, and skills. Chapter 8 provided results on how adopting an experiential learning approach and reflection on technology-mediated language teaching empowered two successful pre-service teachers in New Zealand to describe and critically evaluate their teaching and learning processes and devise plans for future practice, all fostering their autonomy.

Chapter 9 is a descriptive study advocating a more productive view of gaming as an L2 learning activity for increasing learner-player autonomy against the backdrop of “learnful” and “gameful” dispositions of gaming and students' maintaining control and agency over the gaming experience. The penultimate chapter is an auto-ethnographic research as the researcher-participant explored a large number of language apps over a year to learn Spanish to promote her understanding of students' experiences in informal learning contexts. The researcher highlights the need for learners' awareness of app affordances and autonomy because app-based language learning requires learners' continuous learning choices and adapting apps to fine-tune their evolving needs.

The final chapter pertains to an afterword where editors provide novel avenues for research, highlighting the need for studying (1) strategic learning, (2) metacognitive abilities, (3) autonomy in relation to computerized-participation and massive learner data, (4) critical digital literacy and social justice in technology-infused environments, (5) normative behaviors, (re)construction of identities, ideologies, positioning, and power distribution in digital learning spaces, and (6) digital divide; all promoting our autonomous and empowered vision of the learner in digital spaces.

As a language teacher and researcher, I believe the editors and the chapters' authors should be highly praised for their outstanding contributions that offer a plenty of food for thought and pedagogically applicable and theoretically rich insights about the dynamic relation of learner autonomy, language education, and technology to their intended readership; namely, language instructors, researchers, teacher educators, materials/curriculum designers, policymakers, and (under)graduate students. I affirm that the chapters, composed pre-pandemic, enlighten our perspective toward learner autonomy during/beyond pandemic; however, comparing how learner autonomy is understood/enacted in *crisis-prompted imposed* vs. *normal* online education is highly demanded in future research as the former is often characterized by lack of enough preparation, insufficient technological proficiency, unequal access to technology, and structural issues while the latter is normally accompanied by anticipated, accurate planning, extensive investment strategies, and evidence-based practice for planning desired education environments.

Overall, this volume is an eye-opener to how the increasing permeation of technology has blurred the distinction between (lifelong) informal and formal language learning and caused changes in power relationships, technological learning ecologies, enlarged collective knowledge-building, and opportunities for virtual collaboration with computerized/human agents, all signifying the desideratum for placing *learner autonomy* at the center of attention in technology-infused language education research and practice.

## Author Contributions

The author confirms being the sole contributor of this work and has approved it for publication.

## Conflict of Interest

The author declares that the research was conducted in the absence of any commercial or financial relationships that could be construed as a potential conflict of interest.

## Publisher's Note

All claims expressed in this article are solely those of the authors and do not necessarily represent those of their affiliated organizations, or those of the publisher, the editors and the reviewers. Any product that may be evaluated in this article, or claim that may be made by its manufacturer, is not guaranteed or endorsed by the publisher.

